# Modelling person-to-person transmission in an Enterovirus A71 orally infected hamster model of hand-foot-and-mouth disease and encephalomyelitis

**DOI:** 10.1038/emi.2017.49

**Published:** 2017-07-12

**Authors:** Win Kyaw Phyu, Kien Chai Ong, Kum Thong Wong

**Affiliations:** 1 Department of Pathology, Faculty of Medicine, University of Malaya, Kuala Lumpur 50603, Malaysia; 2 Department of Biomedical Science, Faculty of Medicine, University of Malaya, Kuala Lumpur 50603, Malaysia

**Keywords:** Enterovirus A71, hamster model, person-to-person transmission, hand-foot-and-mouth disease, encephalomyelitis, immunohistochemistry, *in situ* hybridization

## Abstract

Enterovirus A71 (EV-A71) causes hand-foot-and-mouth disease (HFMD), which may be complicated by fatal encephalomyelitis. Although fecal–oral or oral–oral routes are important in person-to-person transmission, how viral shedding and exposure may predispose individuals to infection remains unknown. We investigated person-to-person transmission by using a model of HFMD and encephalomyelitis based on EV-A71 oral infection of 2-week-old hamsters. Animals (index animals) infected with 10^4^ 50% cell culture infective doses of virus uniformly developed severe disease four days post-infection (dpi), whereas littermate contacts developed severe disease after six to seven days of exposure to index animals. Virus was detected in oral washes and feces at 3–4 dpi in index animals and at three to eight days after exposure to index animals in littermate contact animals. In a second experiment, non-littermate contact animals exposed for 8 or 12 h to index animals developed the disease six and four days post-exposure, respectively. Tissues from killed index and contact animals, studied by light microscopy, immunohistochemistry and *in situ* hybridization, exhibited mild inflammatory lesions and/or viral antigens/RNA in the squamous epithelia of the oral cavity, tongue, paws, skin, esophagus, gastric epithelium, salivary glands, lacrimal glands, central nervous system neurons, muscles (skeletal, cardiac and smooth muscles) and liver. Orally shed viruses were probably derived from infected oral mucosa and salivary glands, whereas fecal viruses may have derived from these sites as well as from esophageal and gastric epithelia. Asymptomatic seroconversion in exposed mother hamsters was demonstrated. Our hamster model should be useful in studying person-to-person EV-A71 transmission and how drugs and vaccines may interrupt transmission.

## INTRODUCTION

Enterovirus A71 (EV-A71) is a non-enveloped, single-stranded, positive-sense RNA virus of the genus *Enterovirus*, which belongs to the very large *Picornaviridae* family. Apart from coxsackievirus A16 (CVA-16), it is one of the major causes of hand-foot-and-mouth disease (HFMD) and herpangina in young children.^1^ Although EV-A71 infection is usually self-limiting, complications such as aseptic meningitis, acute flaccid paralysis and fatal encephalomyelitis may occur.^2^ Large outbreaks of EV-A71 HFMD with complications have been reported worldwide, mostly in children under five years of age.^3, 4, 5, 6, 7, 8^

Similarly to transmission of other human enteroviruses, such as poliovirus and CVA-16, person-to-person transmission of EV-A71 occurs mainly through fecal–oral and oral–oral routes from infected feces and saliva, as well as via respiratory droplets.^1, 9, 10^ EV-A71 has been consistently isolated from oral secretions, throat swabs, rectal swabs and feces.^1, 11, 12, 13, 14, 15^ The palatine tonsil and oropharyngeal mucosa have been thought to be important primary viral replication sites and to significantly contribute to oral shedding.^16^ To date, there is no evidence that other parts of the human orodigestive tract support viral replication. Viruses shed in the feces may persist for up to six weeks and may be present in throat swabs for up to 24 days.^17, 18, 19^ Skin vesicles in HFMD have been shown to contain viable virus,^11, 14^ and significant viral shedding from the skin may contribute to person-to-person transmission.^1, 20^

There are very few published reports of person-to-person transmission of EV-A71. In one study, it has been found that intra-family transmission usually occurs after direct contact with infected siblings.^12^ A case of very rare, likely adult-onset, acute EV-A71 encephalitis has been reported in a mother presumably infected through close direct contact with one or all her three children, who had previously had uncomplicated HFMD.^21^ On the basis of the results of PCR analysis, her stool was positive for EV-A71; however, her cerebrospinal fluid was negative. EV-A71 was isolated from the feces of all of her children. Therefore, the virus was presumably transmitted through an orofecal route, although no information is available regarding virus isolation from the throat or skin lesions.

In a previous study, 1-day-old mice orally infected by EV-A71 have been found to develop skin lesions and hind limb paralysis and to transmit the infection to their littermate controls; mild skin lesions were observed, but there was no apparent paralysis and/or death in the littermates.^22^ Although fecal/oral secretions were not tested by viral culture or PCR, the authors speculate that transmission may have occurred through feces or close contact with infected animals. To our knowledge, orally infected animal models of EV-A71 infection with demonstrable oral shedding and fecal excretion of virus have not been used to systematically investigate or model person-to-person transmission. The absence of such reports may be because most existing animal models, such as monkey and mouse models, including the AG129 mouse (interferon type 1 and 2 receptor knock-out) and human scavenger receptor class B member 2 (SCARB2) transgenic mouse models, cannot be consistently orally infected.^22, 23, 24, 25, 26^ We have recently reported the successful development and characterization of a hamster model for EV-A71 HFMD and encephalomyelitis that allows for consistent orally infection. This model demonstrates oral shedding and fecal excretion of virus and squamous cell infection in the paws, skin and oral cavity.^27^ In this study, we investigated the potential of this model to model person-to-person transmission of EV-A71 via oral and fecal viral shedding and to help identify the factors that might influence transmission.

## MATERIALS AND METHODS

### Cells and virus stock

Vero cells were grown in Dulbecco’s modified Eagle’s growth medium (Sigma-Aldrich, St. Louis, MI, USA) supplemented with 5% fetal bovine serum (HyClone, Logan, UT, USA). A mouse-adapted EV-A71 strain (MAVS), which has previously been shown to consistently orally infect 2-week-old hamsters, was grown and titrated in Vero cells as previously described.^27^

### Hamster viral transmission study

All animal experiments were approved by the Institutional Animal Care and Use Committee of the Faculty of Medicine, University of Malaya. Pregnant Syrian golden hamsters were obtained from Monash University (Sunway, Malaysia). Unless otherwise stated, all hamsters were housed together with their own mothers until the end of the experiments because 2-week-old hamsters still need to suckle milk, as it is their only food source. In preparation for the animal experiments, preliminary studies were performed to establish the optimal viral dose, cage size and number of infected animals needed per cage to transmit infection efficiently.

### Experiment 1

Five groups of 2-week-old hamsters (*n*=6 per group) from five different mothers were studied to investigate intra-hamster family transmission. From each family group, three animals were orally infected with 10^4^ 50% cell culture infective doses (CCID_50_) of MAVS; these animals represented ‘index case’ hamsters (hereafter called index hamsters). They were then temporarily kept separate from their mothers and the ‘contact case’ littermates (hereafter called contact hamsters) to limit the likelihood of infection by the initial inoculum. After 2 h, index hamsters (*n*=3) were returned to their respective mothers and littermate contact hamsters (*n*=3) and housed together in the same cage (size 20 cm × 30 cm) for the duration of the experiment. Thus, the number of days post-infection (dpi) of index animals and the number of days post-exposure of contact animals (to index animals) began on the same day. Animals were observed several times daily for signs of infection including back hunching, ruffled fur, weight loss and hind limb paralysis. To determine the amount of live virus excretion, oral washes and feces were collected daily from four groups of index and contact animals, as described previously.^27^ Samples were immediately frozen and stored at −80 °C for subsequent virus isolation. In addition, oral wash and fecal samples from four index animals at 4 dpi and from contact animals at eight days post-exposure were titrated for viral titers.

Animals showing severe hind limb paralysis and animals that were moribund were sacrificed by isoflurane inhalation, and sera were collected via cardiac puncture for viral isolation. Carcasses of entire animals from the four animal groups were immediately fixed in 10% neutral buffered formalin for 2 weeks, and tissues were harvested for routine processing. Approximately 20 standard tissue cross-section, paraffin-embedded blocks were prepared as previously described; tissues sampled included tissues from the oral cavity, brain, spinal cord, internal organs, hind limb muscle, paws and skin.^27^ From the 5th group of animals, instead of fixing tissues, we colleted fresh organs/tissues including samples from the brain, spinal cord, stomach and hind limb muscle and immediately froze the tissues at −80 °C for subsequent virus titration along with the collected sera.

### Experiment 2

In a separate experiment designed to study the duration of viral exposure and transmission, three groups of 2-week-old index hamsters (*n*=4 per group; a total of 12 animals) taken from three different hamster families were infected with a dose of 10^4^ CCID_50_ per animal and then returned to their mothers. When the index hamsters in each group developed signs of infection at 4 dpi, they were temporarily separated from their mothers and placed into a clean cage (size 10 cm × 20 cm) together with another group of uninfected 2-week-old contact hamsters (*n*=4) from a different family. In this way, three groups of non-littermate contact hamsters were separately exposed to three groups of index hamsters for 4, 8 and 12 h before they were returned to their mothers. In this experiment, we did not use littermate contact animals because the 2-week-old hamsters needed to be returned to their mothers to suckle; therefore, these animals would come into contact with their infected littermate index hamsters and the exposure period would be prolonged. Index hamsters were sacrificed at 4–5 dpi when they showed severe hind limb paralysis or were moribund. After being returned to their own mothers, contact hamsters were observed and sacrificed when severe disease occurred. Animal carcasses of both index and contact animals in the 12 h exposure group were formalin-fixed for routine processing and examination, as described above.

Samples were collected from all index and contact hamsters studied in this experiment for virus detection. In addition, both samples from index animals and the corresponding 12 h exposed contact animals were virus titrated.

### Virus titration

Oral washes and fecal specimens collected in both experiments were titrated for virus with a standard microtitration assay, as previously described^27^ and PCR. Briefly, all specimens were treated with chloroform (1:10) and centrifuged at 3500 r/min for 20 min at 4 °C; the supernatants were stored at −80 °C for subsequent titration. Oral wash and fecal samples with initial negative results underwent up to two passages to confirm the absence of live viruses. For PCR, total RNA was extracted from samples with a High Pure Viral RNA kit (Roche, Mannheim, Germany). Single-stranded cDNA synthesized with a cDNA synthesis kit (Tetro cDNA Synthesis Kit, Bioline, Landon, UK) was then amplified using a pair of primers as described previously.^27^ The PCR conditions were as follows: initial denaturation at 95 °C for 3 min; 30 cycles of denaturation at 95 °C for 30 s; primer annealing at 53 °C for 30 s; and extension at 72 °C for 1 min; and a final extension at 72 °C for 10 min. PCR products were loaded into 1% agarose gels and stained with ethidium bromide, and the positive 600 bp band was visualized on a UV transilluminator (Alpha Imager HP, South San Francisco, CA, USA).

Virus titer was determined in sera and fresh organs (brain, spinal cord, stomach and hind limb muscles), as previously described.^27^ Briefly, tissues were homogenized in cold PBS to obtain a 10% (wt/vol) suspension, and virus titers were determined in Vero cells using a standard microtitration assay.

### Light microscopy, immunohistochemistry and *in situ* hybridization

Paraffin-embedded tissue blocks (*n*=120 in experiment 1; *n*=128 in experiment 2) were sectioned (4-μm thick sections) and stained with hematoxylin and eosin for light microscopy. Similar sections were prepared for immunohistochemsitry and *in situ* hybridization to detect viral antigens and RNA, respectively, as previously described.^27^

### Neutralizing antibody assay

Serum samples from all mother hamsters from both experiment 1 (*n*=5) and experiment 2 (*n*=6) were collected at sacrifice and tested for neutralizing antibodies. Briefly, the serum samples were serially diluted up to 1:512 and mixed equally with 100 CCID_50_ of EV-A71.^28^ The samples were then incubated at 4 °C overnight before inoculation onto Vero cell monolayers in 96-well plates; the samples were cultured for seven days at 36 °C. Monoclonal mouse anti-EV-A71 antibody was used as a positive control, as previously described.^28^

### Statistics

To determine the significance of differences between mean viral titers, one-way analysis of variance and repeated *t*-test were performed using IBM SPSS Statistics software version 23 (IBM corporation, New York, NY, USA). The results are expressed as the mean±standard deviation. Statistical significance between the percentage of oral wash and fecal viral positivity was determined by one-way analysis of variance. *P* values <0.05 were considered significant.

## RESULTS

### Experiment 1

Index animals orally infected with 10^4^ CCID_50_ of virus in experiment 1 showed typical signs of disease, such as hunched back, ruffled fur, weight loss and hind limb paralysis at 4 dpi, and were killed at 4–5 dpi (Table 1). All littermate contact hamsters showed signs of infection similar to those of index animals after six to seven days post-exposure. This result suggests that contact hamsters showed signs of infection two to three days later than index hamsters. Contact hamsters were sacrificed at seven to 8 days post-exposure.

Virus was detected (up to the 2nd passage) from oral washes in nine index animals at 3-4 dpi (Table 1), in two animals (B1, B2) at 1 dpi, and in 1 animal at 5 dpi (D2). Virus detection in feces was positive in most index animals, specifically, in 6 index animals (A3, B2, C1, C2, C3 and D3), at 4 dpi (range of 3–5 dpi for all animals). Oral positivity was most commonly detected before fecal positivity. Specifically, oral positivity was detected before fecal positivity in seven animals (range of one to three days before), followed by oral and fecal positivity detected on the same day in 4 animals (B3, C1, C2, D2) and fecal positivity detected before oral positivity in 1 animal (A2).

Virus was detected in oral washes from 8 contact animals at three to four days post-exposure and in oral washes from four contact animals (A4, A5, A6 and D4) at 5–8 days post-exposure. Virus was detected in the feces of five contact animals (A6, C4, C6, D4 and D5) at five days post-exposure (range of 4–8 days for all animals). Similarly to that in to index animals, oral positivity in contact animals was most commonly detected before fecal positivity. Specifically, oral positivity was detected before fecal positivity in seven contact animals (range of one to three days before), followed by oral and fecal positivity detected on the same day in four animals (A6, B4, C4, and D5) and fecal positivity detected before oral positivity in one animal (D4). When only positive cultures from the oral wash and feces were counted, oral wash positivity constituted 62%, which was significantly higher than fecal positivity (*P*=0.003).

Thus, in general, the onset of oral virus shedding in the majority of index hamsters started from 3–4 dpi (Table 1). Oral shedding from most contact hamsters started five days post-exposure to index animals. In most index and contact animals, viruses were usually detected later in the feces than in the oral washes, although fecal positivity occurred slightly earlier in index animals.

**Figure 1:**
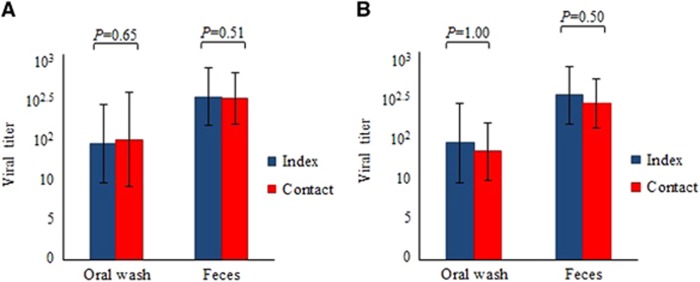
Oral wash and fecal viral titers in index and littermate contact animals (*n*=4 each) at four days post-infection and eight days post-exposure, respectively, from experiment 1 (**A**). Oral wash and fecal viral titers in index animals and non-littermate contact animals (*n*=4 each) in the 12 h exposure group in experiment 2 (**B**). Viral titer is expressed as CCID_50_/mL±standard error of mean per 10% suspension. There were no significant differences between titers; all *P* values were >0.05.

**Figure 2:**
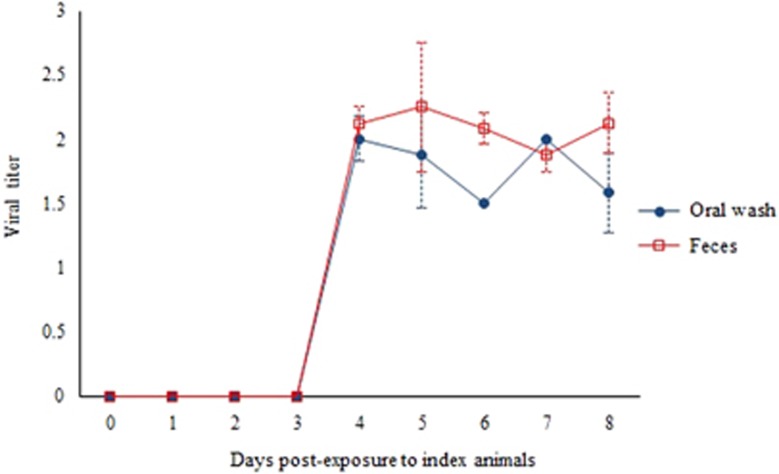
Growth curve of viruses isolated without passage from oral washes and feces of contact animals from experiment 1. Virus titer is expressed as CCID_50_/mL±standard error of mean per of 10% suspension of oral samples and 10% (wt/vol) tissue homogenates of fecal samples.

The mean oral wash viral titers of index animals (*n*=4) at 4 dpi of 4 × 10^1.5^ CCID_50_/mL and of contact animals (*n*=4) at 8 days post-exposure of 3 × 10^1.5^ CCID_50_/mL (Figure 1A) were not significantly different (*P*=0.65). The corresponding mean fecal viral titers of index and contact animals of 1 × 10^2.5^ CCID_50_/mL and 5 × 10^2.5^ CCID_50_/mL, respectively, were also not significantly different (*P*=0.51). Figure 2 shows viral titers in unpassaged cultures of oral wash and fecal samples obtained from contact animals in Experiment 1. For both samples, viral titers were detected at four days post-exposure and maintained until eight days post-exposure to index animals. PCR analysis confirmed the absence of virus in negative oral wash and fecal cultures and confirmed the presence of virus in all positive samples (data not shown).

**Figure 3:**
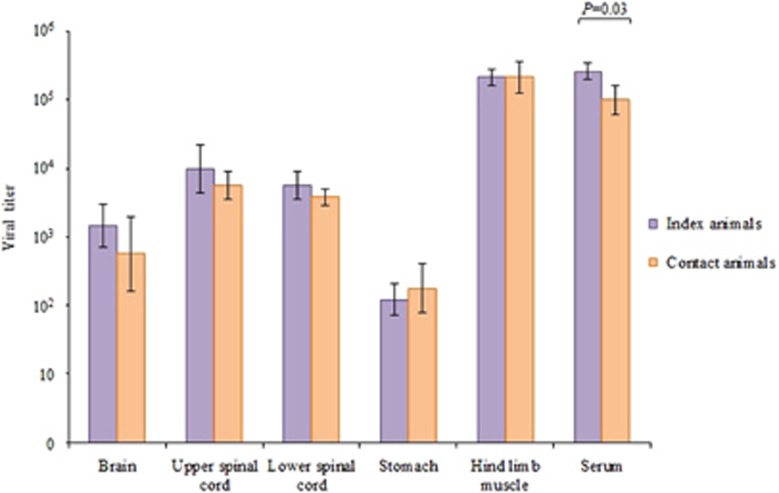
Viral titers from various tissues of index and littermate contact animals (*n*=3 each) in experiment 1 at 4 days post-infection and 8 days post-exposure, respectively. Viral titer is expressed as the mean CCID_50_/mL±sem per 10% tissue homogenate. Viral titer in sera is expressed as CCID_50_/mL±sem per 10% dilution. There were no significant differences between tissue viral titers; *P* values were >0.05, with the exception of sera (*P*=0.03).

Viral titers in brain, spinal cord, stomach and hind limb muscle tissues from index and contact animals (*n*=3 each) from experiment 1 were not significantly different, whereas viral titers in sera from index and contact animals were significantly different (*P*=0.03) (Figure 3).

### Experiment 2

**Figure 4:**
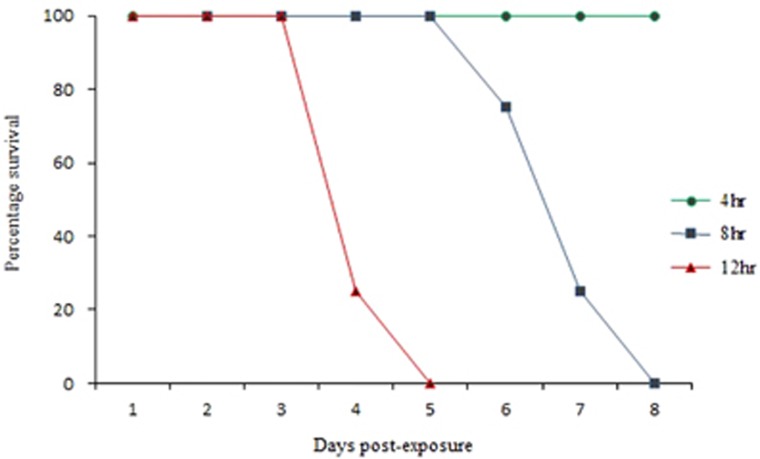
Graphs of ‘survival’ or period before killing of three groups of littermate contact animals (*n*=4 each group) from experiment 2 with 4, 8 and 12 h exposures to infected index animals.

After 4 h of exposure to four severely infected index hamsters, the four non-littermate contact hamsters, after being returned to their mothers, remained healthy for up to 14 days post-exposure (Table 2, E5–E8). In the 8 h exposure group, all four non-littermate contact animals (F5–F8) showed signs of infection, such as hunched back, ruffled fur, weight loss and hind limb paralysis at 6 days post-exposure, and were killed at 6–8 days post-exposure. After 12 h of exposure to index animals, all four non-littermate contact animals (G5–G8) developed severe disease earlier at four days post-exposure and were killed at four to five days post-exposure. This direct relationship of a longer duration of exposure of contact animals to index animals resulting in a shorter duration of ‘survival’/period before killing is summarized in Figure 4.

In the four non-littermate, contact animals with 4 h of exposure (E5–E8), oral or fecal virus was not detected between one and 14 days post-exposure, a result consistent with observations that animals did not show any signs of disease. In the 8 h exposure group, the result of virus isolation from oral washes was positive in all four contact animals (F5–F8) and from feces was positive in two animals (F5, F6) at six to seven days post-exposure. In the 12 h exposure group, virus in oral washes and feces from all four contact animals (G5-G8) was detected four to five days post-exposure (Table 2). Thus, as expected, virus was more likely to be detected in both the oral wash and feces of contact animals in the 12 h exposure group.

All index animals developed severe disease, as described in experiment 1, and were killed at 4–5 dpi, as previously described (Table 2). Virus was isolated from the oral washes of 11 index animals (Table 2) at 4–5 dpi, whereas no virus was isolated from the oral wash of one animal (F2). Fecal virus was positive in eight animals at 4–5 dpi and negative in four animals (E2, F3 G3 G4). Of the 7 animals in which both oral and fecal virus were positive, virus was detected on the same day.

Mean oral wash viral titers of index animals (*n*=4) at 4–5 dpi of 8 × 10^2^ CCID_50_/mL and of contact animals (*n*=4) at four days post-exposure of 3 × 10^2^ CCID_50_/mL (Figure 1B) were not significantly different (*P*=1.00). Similarly, the corresponding mean fecal viral titers of index and contact animals at 2 × 10^3^ CCID_50_/mL and 1 × 10^3^ CCID_50_/mL, respectively, were also not significantly different (*P*=0.50).

### Pathological findings

**Figure 5:**
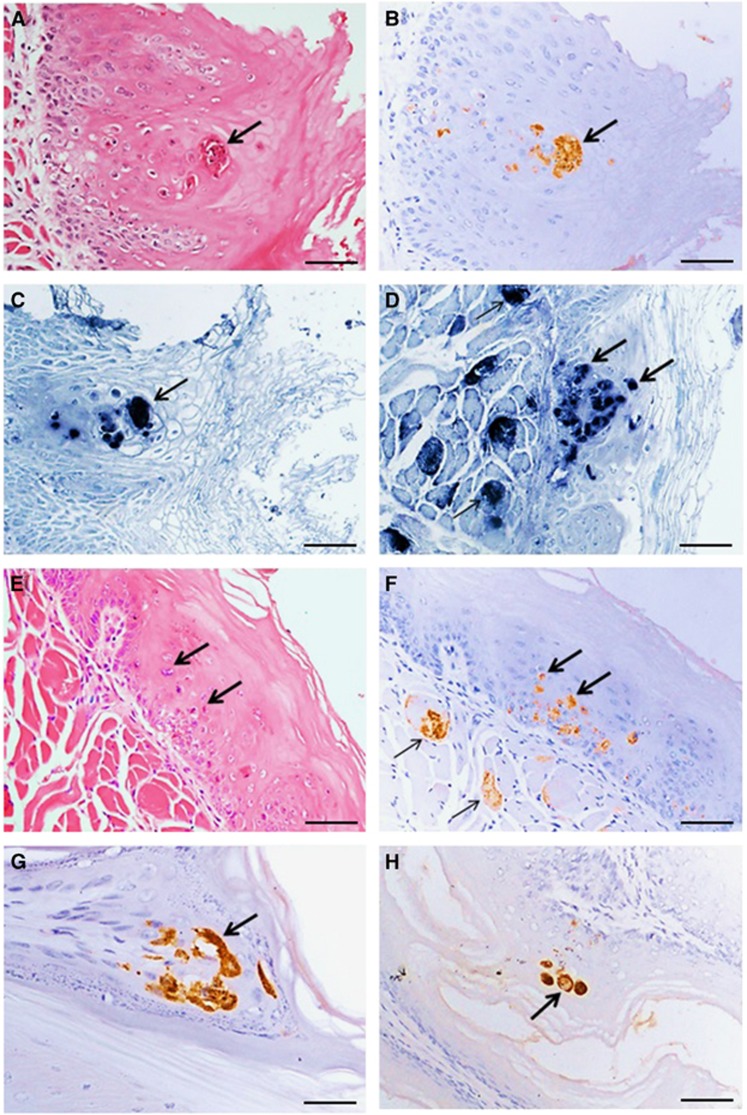
Pathological findings in squamous cells and skeletal muscles in littermate contact hamsters at 8 days post-exposure (experiment 1). Squamous epithelial cell necrosis in the oral mucosa (**A**); localization of viral antigens (**B**) and viral RNA (**C**) in the same lesion in adjacent tissue sections. A mildly inflamed lesion in tongue squamous epithelial cells (**E**) has viral antigens in the same lesion (**F**) and skeletal muscle fibers (**F**) in adjacent tissue sections, as well as viral RNA in the same tongue squamous lesion (**D**) and skeletal muscle fibers (**D**). Viral antigens in squamous cells in paw epidermis (**G**) and esophageal mucosa (**H**). Stains: hematoxylin and eosin (**A**,**E**) immunohistochemistry with 3, 3’ diaminobenzidinetetrahydrochloride chromogen/hematoxylin (**B**,**F**,**G**,**H**) and *in situ* hybridization with nitroblue tetrazolium/5-bromo-4-chloro-3-indolyl phosphate/hematoxylin (**C**, **D**). Original magnification: 20x objective (**A–F**,**H**), × 40 objective (**G**). Scale bars: 30 μm (**A–F**,**H**), 15 μm (**G**).

**Figure 6:**
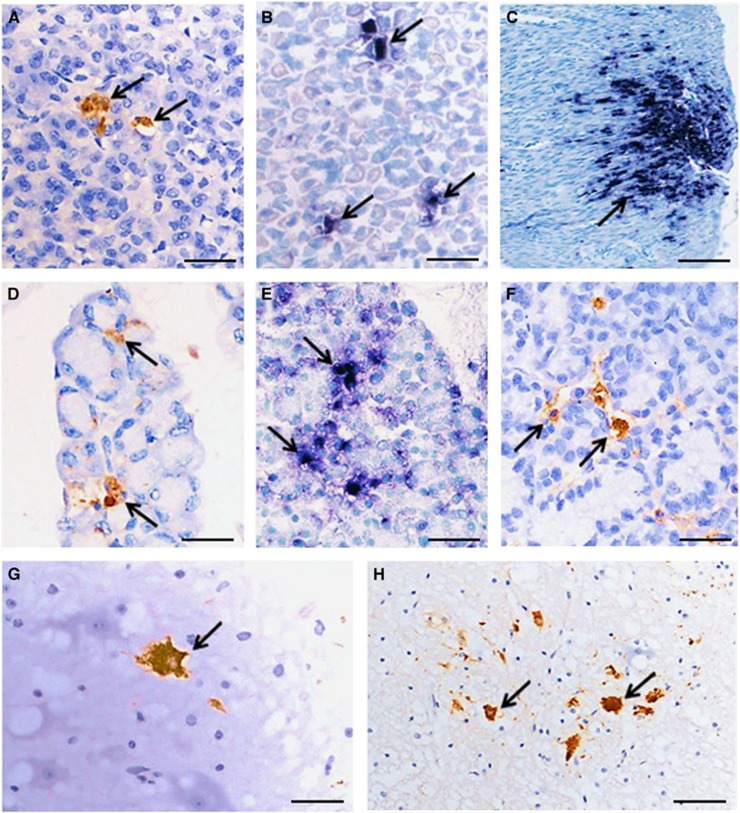
Pathological findings in the orodigestive tract and central nervous system in littermate contact hamsters at 8 days post-exposure (experiment 1). Viral antigens (**A**) and viral RNA (**B**) in gastric mucosal epithelium, and viral RNA in gastric smooth muscle (**C**). Viral antigens (**D**) and viral RNA (**E**) were detected in salivary gland acinar cells. Viral antigens in lacrimal gland acinar cells (**F**), brainstem neurons (**G**) and spinal cord anterior horn cells (**H**) were also detected. Stains: Immunohistochemistry with 3, 3’ diaminobenzidinetetrahydrochloride chromogen/hematoxylin (**A**,**D**,**F–H**) and *in situ* hybridization with nitroblue tetrazolium/5-bromo-4-chloro-3-indolyl phosphate/hematoxylin (**B**,**C**,**E**). Original magnification: 10x objective (**C**), × 20 objective (**H**), and × 40 objective (**A**,**B**,**D–G**). Scale bars: 50 μm (**C**), 30 μm (**H**), and 15 μm (**A**,**B**,**D–G**).

Macroscopic skin lesions around the oral cavity and on the paws of some infected animals were observed (data not shown). In the 14 severely infected animals (experiment 1: index and contact animals, *n*=3 each; experiment 2: index animals and contact animals, *n*=4 each), pathological findings were generally similar (Figures 5 and 6). Viral antigens/RNA were localized in the squamous mucosa in the oral cavity, tongue, esophagus, epidermis (paw and skin), gastric mucosa, central nervous system neurons, muscle (skeletal, cardiac and smooth), lymphoid tissues, liver, salivary glands and lacrimal glands. Viral antigens/RNA appeared to be most widespread in the skeletal muscles, oral mucosa and tongue. Inflammation was absent or minimal in most infected tissues.

### Neutralizing antibody

All mother hamsters from experiments 1 and 2 showed no signs of disease. Sera from killed mother hamsters in experiment 1 (*n*=5) collected at a mean of 22 days post-exposure (range of 18–28 days) had neutralizing antibodies of 1:256. In experiment 2, hamster mothers (*n*=3) of index animals, which were sacrificed at 19 days post-exposure, also developed neutralizing antibodies of 1:256. The hamster mother of the 4 h exposure contact animal group (Table 2) did not show seroconversion at 28 days post-exposure. However, the hamster mothers in the 8 and 12 h exposure groups both had neutralizing antibodies of 1:256 at 22 and 19 days post-exposure, respectively. Thus, all mother hamsters exposed to animals that showed signs of disease seroconverted. In the 4 h exposure group, contact animals (*n*=4) did not exhibit seroconversion.

## DISCUSSION

The orally infected hamster model used in this study is unique because the hamsters consistently develop disease reminiscent of HFMD and encephalomyelitis. Moreover, the pathology closely resembles findings in human autopsies.^27^ Our results showed that this model can also be used to model person-to-person EV-A71 transmission, although viral transmission in human populations is influenced by many complex inter-related factors. To our knowledge, neither a reliable orally infected animal model for EV-A71^27^ nor a model for person-to-person transmission has been previously described. In a mouse model involving EV-A71 oral infection in 1-day-old mice, viral transmission to littermates has been observed. However, the animals develop only very mild skin lesions and survive. Although the authors of that study did not attempt to isolate virus in oral fluids or feces, they have suggested that fecal–oral transmission occurred.^22^ In another study involving orally infected 7-day-old mice, viruses have been detected in the stools of healthy littermate animals, thus suggesting oral–fecal viral transmission; however, no further detailed investigations have been carried out.^29^ In a pig model involving oronasal and orally infected gnotobiotic, only very mild fever has been observed, and rectal swabs from infected animals have been found to be positive for virus.^30^ A promising SCARB2 (a well-recognized viral receptor) transgenic mouse model for EV-A71 infection has been reported but unfortunately cannot be consistently orally infected.^24^

In experiment 1, both index and littermate contact hamsters developed similar signs and pathology of disease. However, contact animals consistently developed disease only after six to seven days post-exposure to index animals, probably because the index animals began to shed the virus from 3 dpi onward. Moreover, littermate contact animals may have been exposed to viruses found in oral fluids, feces and/or the environment in doses that may have been relatively lower than the initial doses administered to index animals. Nonetheless, the incubation period for both index and contact animals was similar, approximately three to four days.

Experiment 2 showed that 8 and 12 h of exposure to index animals successfully enabled viral transmission to non-littermate contact animals, whereas the 4 h exposure group remained healthy with no seroconversion. As expected, the 12 h exposure group developed disease earliest. Interestingly, after 4 days post-exposure, the 12 h exposure group developed severe disease, similarly to index animals, thus suggesting that a relatively short exposure to active infective sources (four index animals) was sufficient for viral transmission to lead to severe infection. Although the cumulative infective doses received by severely infected littermate and non-littermate contact animals were unknown, our results showed that oral and fecal virus titers from index animals of (3–8) × 10^2^ CCID_50_/mL and (1–2) × 10^3^ CCID_50_/mL, respectively, were sufficient for viral transmission. These results correlated with the previous findings in the hamster model in which oral administration of viral doses of 10^2^ to 10^3^ CCID_50_ have been found to be sufficient to cause severe disease.^27^

In most index and contact animals in experiment 1, the onset of oral virus shedding was from 3 to 4 dpi or five days post-exposure. However, in most animals, oral wash viruses were usually detected earlier than were fecal viruses. In the orodigestive tract, viral antigens/RNA were consistently found to be most abundant in the oral mucosa and tongue, and were detected more focally in the salivary glands, lacrimal glands, esophageal epithelia and stomach epithelia. Importantly, viral antigens/RNA were not detected in other parts of the orodigestive tract, including the intestines. These results suggested that, in the hamster model, the main source of oral viral shedding was the oral cavity, and possibly to a lesser extent, the salivary gland. We speculate that fecal virus may be mainly derived from viruses in swallowed oral secretions, with some contribution from viral replication in the esophageal and stomach epithelia. Because EV-A71, poliovirus and other enteroviruses^1, 31^ are able to resist gastric acid, virus from the oral cavity, esophagus and stomach may remain viable when they are excreted in the feces. This viability may be a reason why fecal viruses were detected relatively later than oral viruses. However, viral titers from oral wash and feces cannot be directly compared, because of differences in sample collection. Nonetheless, the percentage of positive oral viral isolation was higher at 62% than fecal positivity at 38% (*P*=0.003), a result consistent with findings from human studies in which throat swabs have been found to be more likely to be positive for virus than rectal swabs or stools.^14^ In humans, the reason for this phenomenon is uncertain but has been postulated to be that palatine tonsils and oral mucosa are major viral replication sites.^16, 20^ Hamsters do not have tonsils.

After exposure to infected offspring, seroconversion without any sign of disease suggested that mother hamsters may have had the human equivalent of asymptomatic infection. We have also found that hamsters older than four weeks did not develop disease after infection (unpublished data), thus providing another potential reason why mother hamsters showed no signs of disease. It has been observed that all family members may be susceptible to infection through close contact with EV-A71-infected patients, but may or may not develop overt signs of infection.^12, 32^ In addition, seroconversion in adults have been found to be >50%, and seropositive rates among family members may be as high as 93%.^12, 32^ The intrafamily transmission rate among family members, especially between siblings, has been reported to be highest.^32^ It has also been suggested that, in some circumstances, asymptomatic or mildly symptomatic adults/parents may be a source of infection for young children. Interestingly, adults may even develop encephalomyelitis after viral transmission from children who experienced only the milder HFMD, thus suggesting that other factors, including a higher infective viral dose and the host immune system, may be responsible for neurovirulence.^12, 15, 19, 21^ In a model involving 7-day-old mice, non-neutralizing, anti-EV-A71 antibodies have been detected in the dams of infected animals and control littermates, although the animals remained healthy.^29^

In conclusion, our data confirmed that oral–oral and oral–fecal routes are important in person-to-person EV-A71 transmission leading to viremia and CNS infection. This hamster transmission model should be a useful model for understanding natural viral transmission and transmission rates in the human population. In addition, it may even be possible to study viral genetic diversity and mutations after transmission over several cohorts of animals. This model may also be used to test whether certain anti-viral agents, particularly orally administered agents, could be used to decrease oral cavity viral replication and excretion and/or inactivate excreted viable viruses in the oral cavity. Theoretically, suitable and safe oral anti-viral agents that act in these ways could greatly affect person-to-person transmission and control of HFMD epidemics.

## Figures and Tables

**Table 1:**
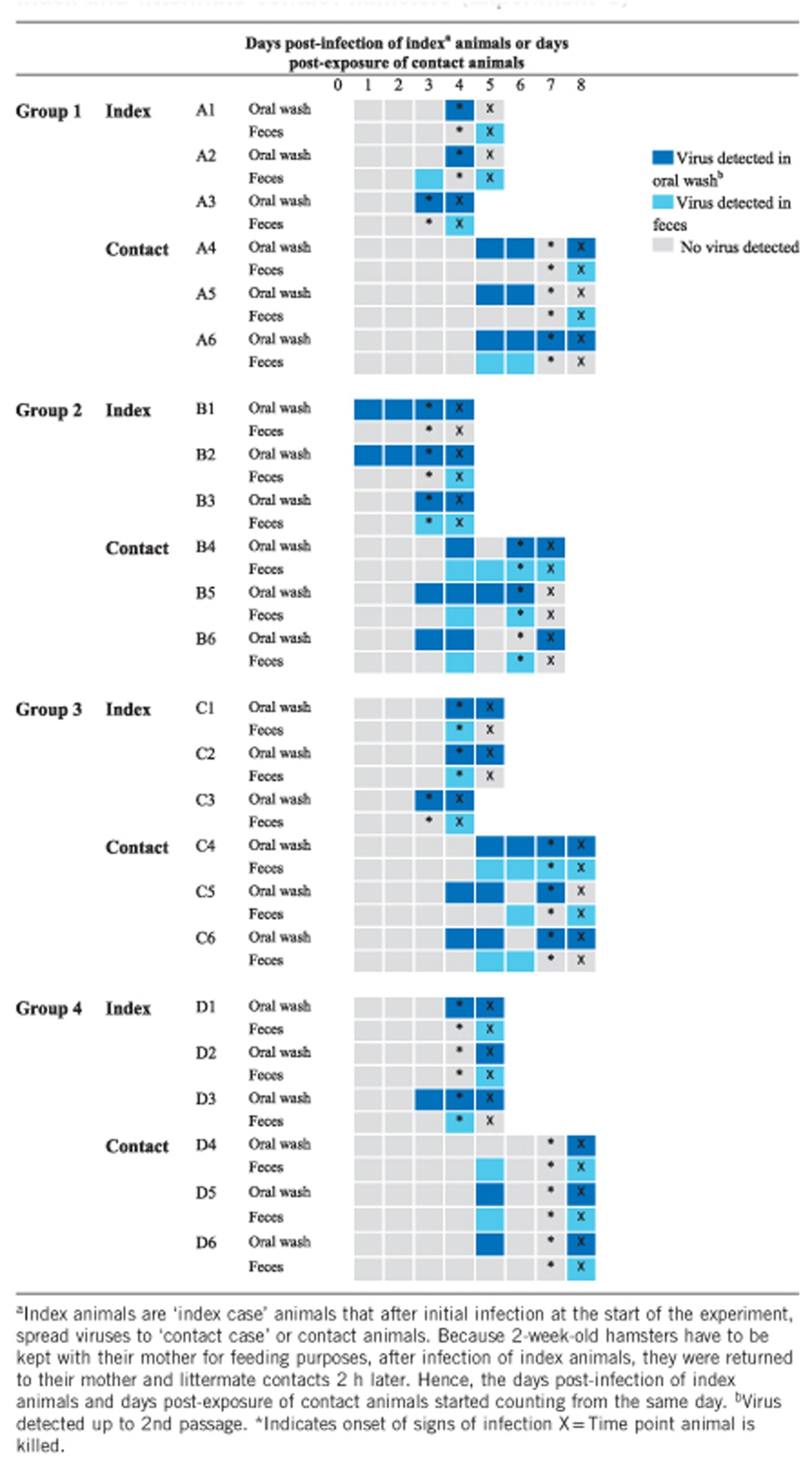
Virus isolation from oral washes and feces in 4 groups of index and littermate contact hamsters (Experiment 1)

**Table 2:**
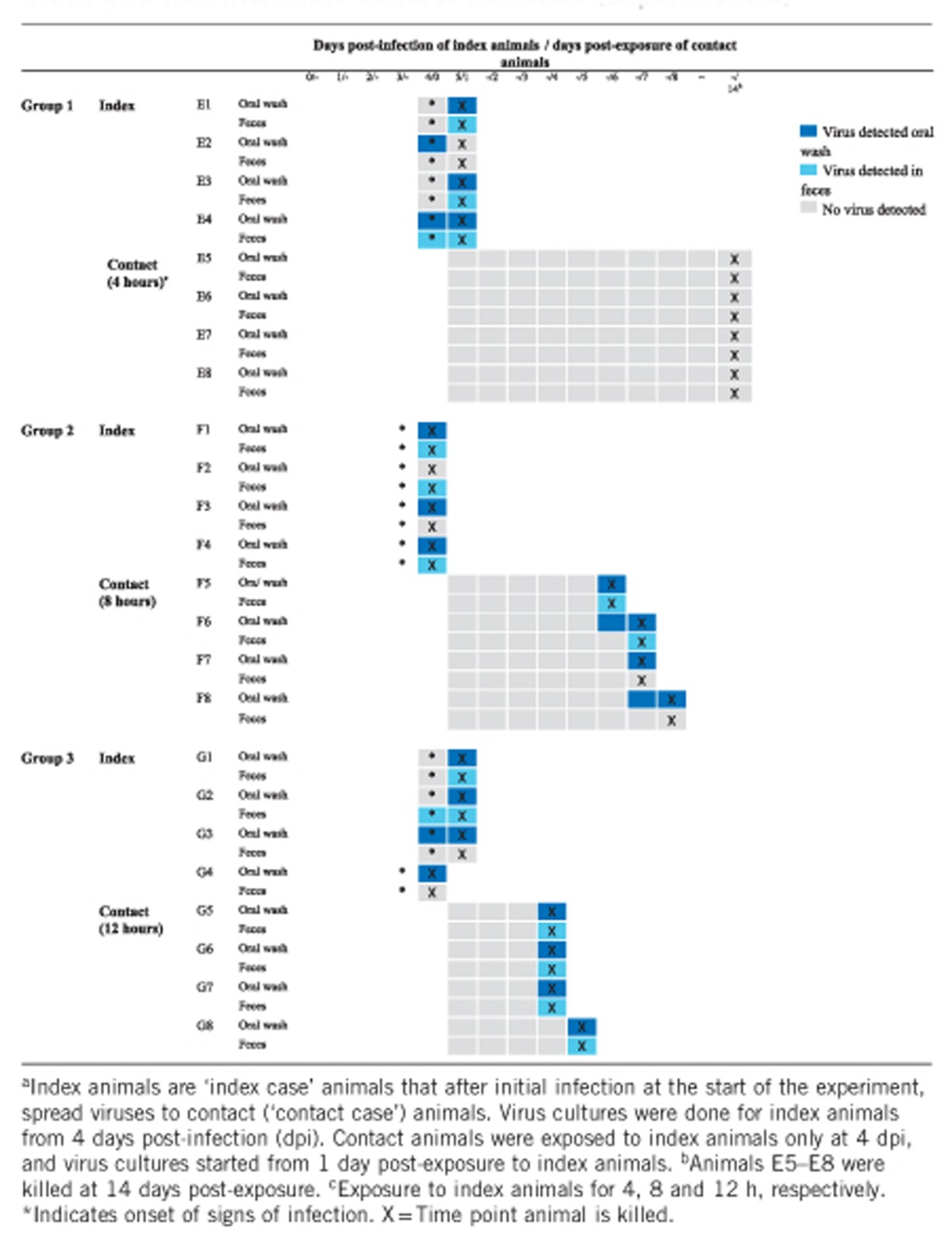
Virus isolation from oral washes and feces in three groups of index and non-littermate contact hamsters (Experiment 2)
